# An anatomical investigation of the proximal vertebral arteries (V1, V2) in a select South African population

**DOI:** 10.1007/s00276-021-02712-x

**Published:** 2021-03-10

**Authors:** B. R. Omotoso, R. Harrichandparsad, I. G. Moodley, K. S. Satyapal, L. Lazarus

**Affiliations:** 1 Department of Clinical Anatomy, School of Laboratory Medicine and Medical Sciences, College of Health Sciences, University of KwaZulu-Natal, Westville Campus, Private Bag X54001, Durban, 4000 South Africa; 2 Department of Neurosurgery, School of Clinical Medicine, College of Health Sciences, Nelson R Mandela School of Medicine, University of KwaZulu-Natal, Durban, South Africa; 3 Department of Radiology, Jackpersad and Partners Inc, Specialist Diagnostic Radiologists, Lenmed Ethekwini Hospital and Heart Centre, Durban, South Africa

**Keywords:** Vertebral artery dominance, Tortuosity, Vertebral artery hypoplasia, Arch of aorta, Iatrogenic injury, Anatomical variation

## Abstract

**Introduction:**

The most common type of vascular complication during cervical spine surgery is the vertebral artery (VA) injury. The presence of anatomical variation in the artery's morphology has been a significant factor for arterial injury during surgery. Therefore, physicians planning interventions in the craniospinal region need to be aware of the extents of variations. In addition to vascular injury, anatomical variations can predispose to some pathologies in the posterior circulation territory. To provide useful data to interventional radiologists, anatomists, and surgeons, we evaluated the anatomical features of the V1 and V2 segments of the VA in a South African population.

**Materials and methods:**

The study is an observational, retrospective chart review of 554 consecutive South African patients (Black, Indian and White) who had undergone computed tomography angiography (CTA) from January 2009 to September 2019.

**Results:**

The VA exhibited morphological variation in its course. We report the incidence of variant origin of the left VA, all from the aortic arch. Variation in the level of entry into the transverse foramen ranged between C7 and C3. A left dominant pattern was observed; we also report on hypoplasia of the VA. In addition, we report incidence of VA tortuosity at V1, V2 to be 76.6% and 32.1%, respectively.

**Conclusions:**

The baseline data established in this study regarding the diameter, variant origin, and level of entry into the transverse foramen will assist neurosurgeons and interventional radiologists in interpreting, diagnosing, and planning and executing various vascular procedures and treatment of pathology in the vicinity of the VA.

## Introduction

Vertebral artery (VA) injury is the most common type of complication in cervical spine surgery [8]. A recent meta-analysis has revealed that patients with variant anatomy are more prone to iatrogenic injury of the VA [17]. Other authors also confirmed that the presence of anatomical variations in the morphology of the VA increases the likelihood of injury [8]. Therefore, variations in the origin and course of the artery should be considered by vascular surgeons and radiologists working in the craniospinal region. A possible explanation for this may be that variant arteries are often situated in an unanticipated position. In addition to the risk of injury, anatomical variations have been associated with some pathologies in the posterior circulatory territory. Important central nervous system structures such as the cervical spinal cord, brainstem, thalamus, cerebellum, and occipital lobes are primarily supplied by the VA [36]. Sometimes, morphologic variations may influence the hemodynamics of blood flow to these central nervous system structures. Inadequate perfusion of these structures can predispose to some pathological process in the posterior circulation territory of the brain.

Authors have hypothesized that the incidence of posterior circulation infarctions of the posterior inferior cerebellar and basilar artery territories is higher in VA dominant patients [44]. Similarly, posterior circulatory stroke and VA occlusion are found to be related with VA hypoplasia [31]. Other previously reported variations include variant origin and duplicate or triplicate origin, variation in the level of entering the transverse foramen (TF) (between C7 and C3), fenestration, and presence of tortuosity. According to some researchers, prevalence of variation sometimes can be linked with anthropometric parameters, demographic and ethnic/racial differences [9, 12]. There have been controversies regarding the cut-off value for hypoplastic VAs and VA dominance in the literature [11]. There was no consensus with regard to which side is dominant in several populations. Some authors have reported left dominance [1, 28] whilst others reported right [11]. However, Mitchell (2004) reported no difference (codominance) [30]. For these reasons and considering the multiracial background of the South African population, there is a need for data on the morphology of the proximal VAs to describe the trend of variation in the local population group. Population-specific data will be more appropriate as data from one population group may not be applicable to another.

VAs are large major arteries of the neck which have their origin from the supero-posterior aspect of the first part of the subclavian artery [15]. It is divided into four segments: the first segment (V1) extends from the origin at the subclavian artery to the C6 transverse process. The second segment (V2) extends from C6 to C2 transverse processes. The third segment (V3) extends from C2 to the site of passage through the foramen magnum. The intracranial segment (V4) extends from the foramen magnum dura to the vertebrobasilar junction [6]. Proper identification of variant anatomy during preoperative planning can reduce the risk of iatrogenic injury. Inadequate information about variation can expose the VA to the risk of injury resulting in grave consequences, especially if the dominant artery is involved in asymmetry [17].

In this retrospective observational study, using images produced by multidetector computed tomography angiography (MDCTA), we sought to investigate the anatomical variations of the V1 and V2 segments of the VA and their clinical relevance for procedures in the vicinity of this part of the artery in a select South African population. Due to the multiracial composition of South African population, in addition to overall incidence of variation, we also report variations based on the three racial groups present: Black, Indian, and White South African.

## Materials and methods

### Study population

This study was a retrospective observational chart review of 554 radiographic images, MDCTA of the extracranial segments (V1, V2) of the VAs to establish the variations that may be present in the morphology and the morphometry of the artery. The design was approved by the Institutional Review Board/Ethics Committee (Biomedical Research Ethics Committee of the University of KwaZulu-Natal with ethical No: BE 148/19). We retrospectively identified 554 CTAs obtained from Lenmed Ethekwini Hospital and Heart Centre, Durban, South Africa, from January 2009 to September 2019.

The angiographies were of 307 males (55.4%) and 247 females (44.6%). The average age of the patients is reported as median (IQR): 62 (23) (range 10–99) years; 62 (25) for female patients and 61 (23) for male patients. Race was defined according to the guidelines outlined in the modern systems of racial classification in the Republic of South Africa [23]. The criteria used in the scheme of racial classification include skin color and ancestry. The South African population is divided into four main racial groups: White, Black, Indian, and Colored. Three population groups were included in the present study: Black 91 (16.4%), Indian 176 (31.8%), and White 287 (51.8%). According to the modern system of classification, a White individual was defined as a person of European descent. A Black individual was defined as a person having origins in any of indigenous Africa or Native group. An Indian individual was defined as a person of Asian descent [23].

Images were analyzed using a Picture Archiving Communication system (PACS). The 3D-MDCTA images were examined for vascular variations by a neurosurgeon, a neuroradiologist, and an anatomist using the coronal and sagittal view. Exclusion criteria included CTA scans that showed no clarity of the VA’s course, scans that showed any sign of damage to the vertebral bones and scans with motion artifacts or poor-quality imaging. Cases of variant origin were analyzed differently and together with the typical group.

### CTA imaging protocol

The imaging examination was performed on a 64-detector row computed tomography (CT) scanner (Lightspeed CT, GE Healthcare Medical Systems, Milwaukee, WI, USA) with the scanning protocol as follows: 120 kVp, 697 mAs, beam collimation 64 × 0.625 mm, gantry rotation time 0.4 s, section thickness of 0.625 mm, pitch 0.969:1 and reconstruction interval of 0.625 mm. During the procedure, infused 80 mL of non-ionic iodinated contrast was followed by 40 mL saline and injected via a double power injector (Medex flowSens, Geubert USA) into the patient’s antecubital vein (4 mL/s).

### Imaging reconstruction

Postprocessing of three-dimensional images was performed by using a multiplanar reformation (MPR), maximum intensity projection (MIP), multiplanar reconstruction (MPR), and volume rendering (VR) algorithms. The volumetric MDCTA data sets were processed on Advanced Workstation 4.2, (GE Healthcare, Milwaukee, USA). The CTAs were performed for diagnostic purposes in the context of various cerebrovascular accidents or diseases. In some cases, the suspected diseases were not found on CT angiography; thus, some materials in this study were derived from a healthy population.

**Fig. 1:**
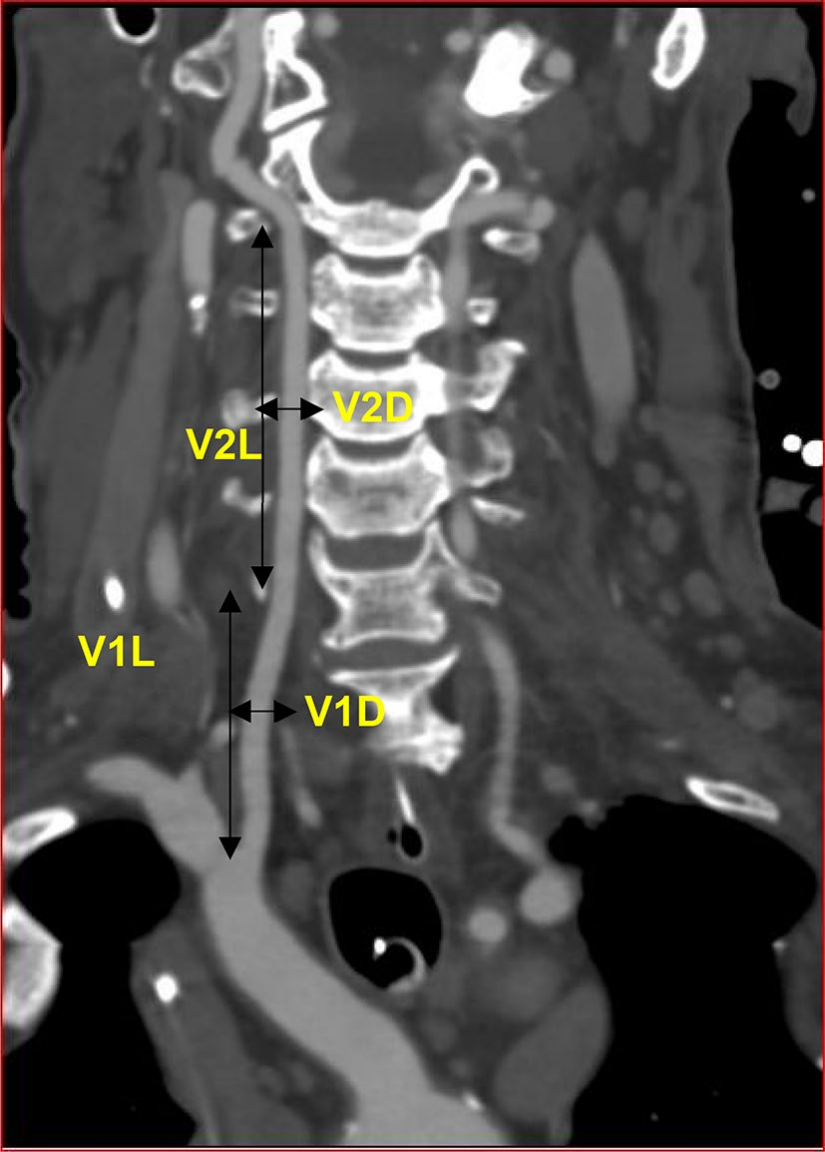
Coronal view of CTA image, V1 and V2 segments of the vertebral artery. Measured variables; V1L—the length of the VA from the origin to the point of entry into the transverse foramen; V2L—the length of the VA from the point of entry into the transverse foramen to the transverse foramen of the C2 cervical vertebra; V1D—the diameter of the V1 segment at a midpoint between origin and point of entry into the transverse foramen; V2D—the diameter of the VA V2 segment at a fixed point between C4 and C3 pedicles

Each radiological image was evaluated for the following parameters, and variables were recorded:

1. Origin of the bilateral VA.
2. Level at which each of the VA entered the TF.
3. Tortuosity at V1 and V2 segment if present. When the artery was tortuous, the software was used to straighten it, after which the length was measured.
4. Total length of the V1 segment from the origin to the point of entry into the TF and the V2 segment, as measured from the point of entry into the TF to the TF of the C2 cervical vertebra (Fig. 1).
5. Diameter of the V1 segment, measured at a midpoint between origin and point of entry into the TF. The diameter of V2 segment was measured at a fixed point between C4 and C3 pedicles. The VA was categorized as dominant if the diameter was significantly larger than the contralateral side by any size [34]. Each VA with a diameter of less than 2.7 mm was noted and classified as hypoplastic [12].

The accuracy and repeatability of the measurements were determined by random sampling of 55 scans, and a second observer took measurements for inter-observer reliability testing.

### Statistical analysis

Statistical analysis was conducted using SPSS version 27 (SPSS Inc., Chicago, IL, USA), and p-values less than 0.05 were considered statistically significant. The normal distribution of continuous variables was tested with the Kolmogorov–Smirnov test. Because the continuous variables are not normally distributed, Wilcoxon Signed Rank test was used to compare paired samples. Kruskal–Wallis test was used to determine statistically significant differences in the dependent variables between the three racial groups and the Chi-square test was used for categorical variables. The continuous variables are presented as median (interquartile range), and the categorical variables were represented by a number (N) and percentage. The Interclass coefficient correlation was used to examine the reliability of measurements.

## Results

The interclass coefficient correlation for intra-observer reliability testing was 99% for the V1 length, and 96% for diameter, 96% for the V2 length, and 95% for the V2 diameter. For inter-observer reliability testing, the intraclass correlation was 85% for the V1 length and diameter, 87% for the V2 length and 85% for the V2 diameter, with a 95% confidence interval.

### Vertebral artery origin

**Fig. 2:**
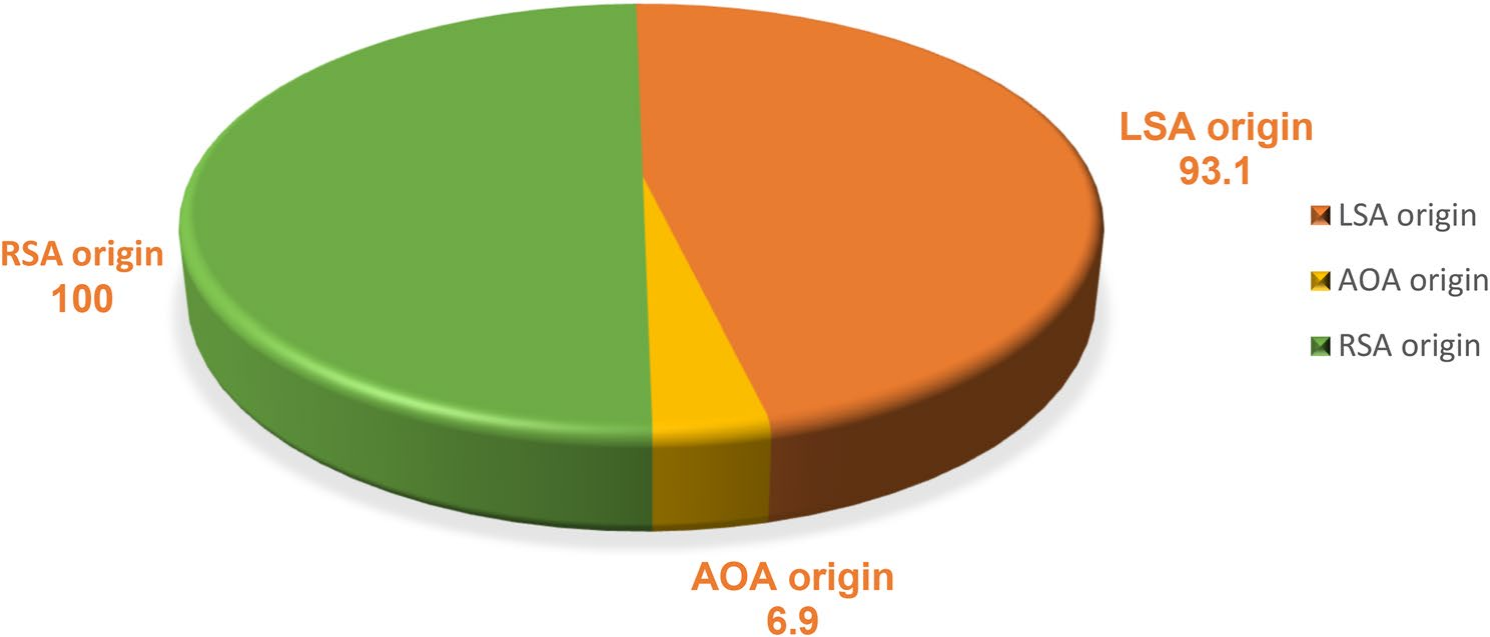
Pie chart of the frequency (percent for each laterality) of variation in origin. LSA: left subclavian artery, *RSA* right subclavian artery, *AOA* arch of aorta.

Much of the VA showed typical origin from the subclavian artery bilaterally in this study. The left VA arose directly from the arch of aorta (AOA) in 6.9% of the patients (38/554: 4.0% males and 2.9% females) (Fig. 2).

**Fig. 3:**
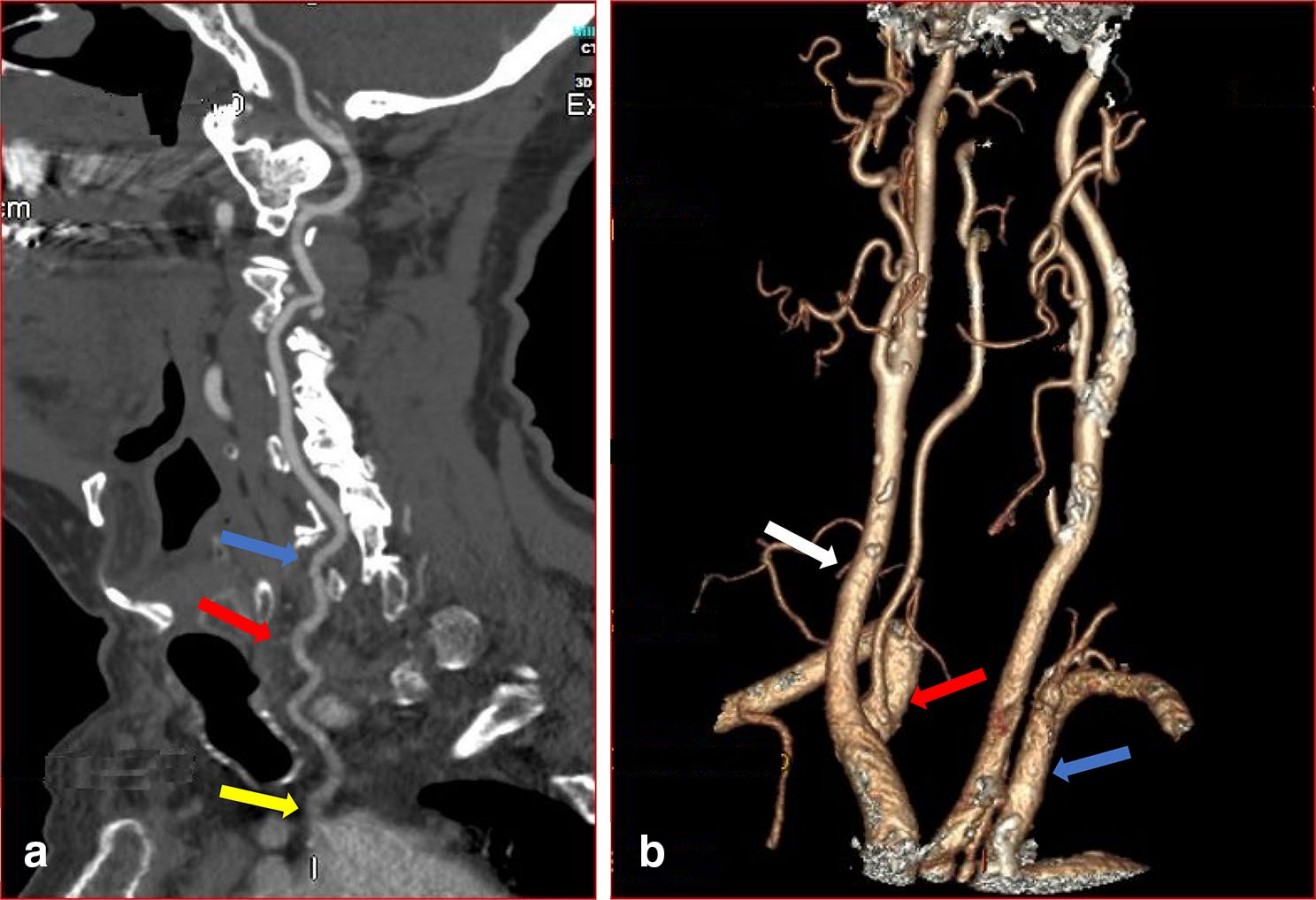
CTA images of a male (sagittal view a) and female (3D reconstructed image b) patients showing variation in origin, level of entering the transverse foramen and tortuosity at V1 segment. **a** The left VA (yellow arrow) originates directly from the AOA with multiple loops (red arrow) and enters through the transverse foramen of C5 vertebra. (blue arrow). **b** 3D reconstructed image shows origin of the right VA from the right subclavian artery close to the bifurcation of the brachiocephalic trunk (red arrow). The right common carotid artery (white arrow) and left subclavian artery (blue arrow) are illustrated (color figure online)

Of these total variations in origin registered, 3.6% were White, 2.5% Indian, and 0.7% Black South Africans. All the right VAs took their origin from the subclavian artery, although two of the right VAs arose close to the bifurcation of the brachiocephalic artery (Fig. 3b). No significant racial or gender differences were noted in the site of origin of the VA (*p* = 0.54, *p* = 1.0).

### Level of entry into the TF

In most cases, the first segment of the VA entered the TF of C6 vertebra (Table 1). On both sides including the variation group (AOA origin), atypical entrance level was most common at the TF of C5.

**Table 1:** Relationship between VA origin and level of entry the TF

Entry level	LVA		RVA
	LSA origin *n* (%)	AOA origin *n* (%)	RSA origin *n* (%)
C3	–	–	1 (0.2%)
C4	3 (0.6%)	5 (13.2%)	9 (1.6%)
C5	16 (3.1%)	31 (81.6%)	40 (7.2%)
C6	489 (94.8%)	1 (2.6%)	502 (90.6%)
C7	8 (1.6%)	1 (2.6%)	2 (0.4%)

*LSA* left subclavian artery, *RSA* right subclavian artery, *AOA* arch of aorta, *LVA* left VA, *RVA* right VA

### Tortuosity

**Fig. 4:**
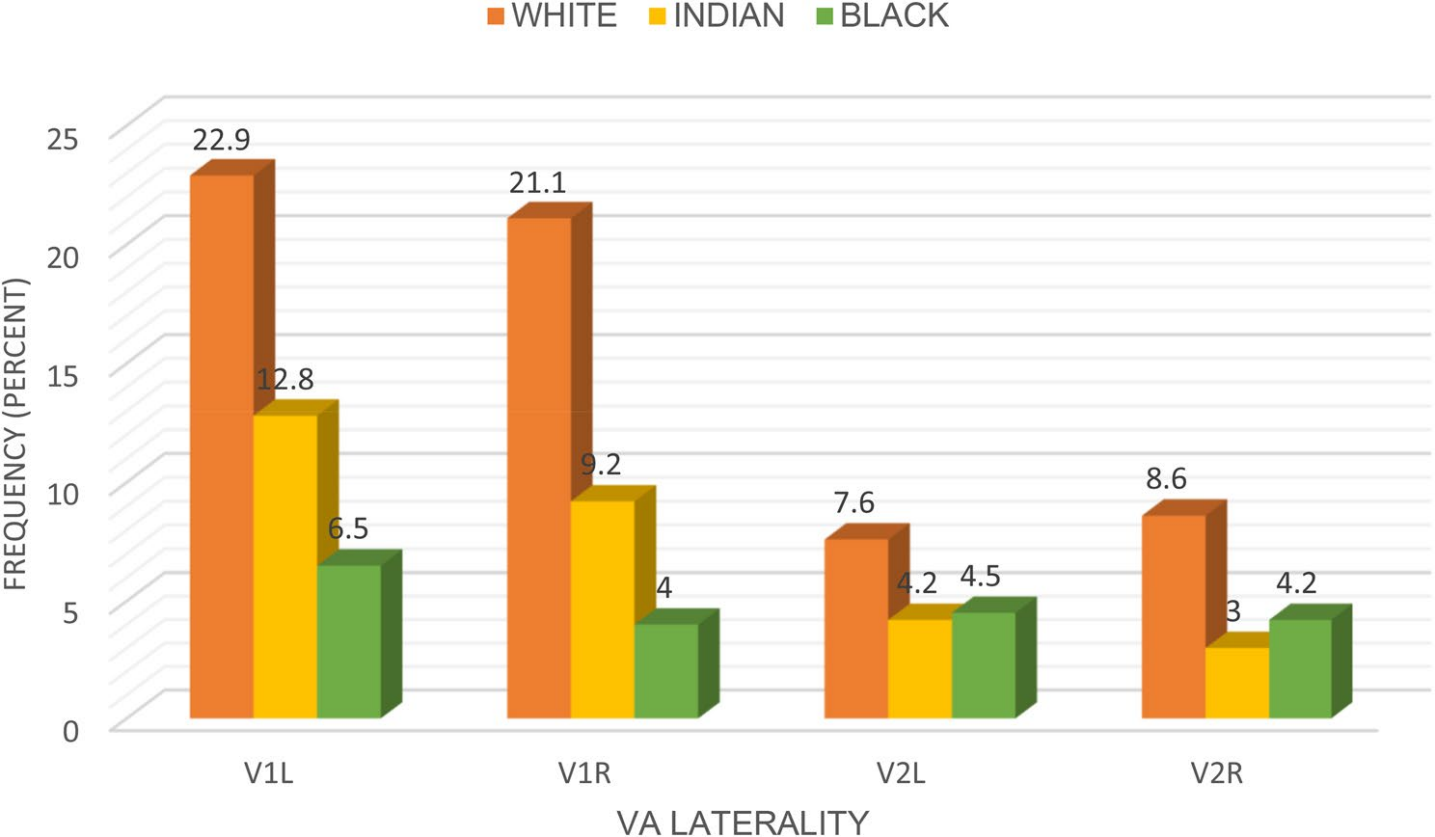
Bar chart of the incidence of tortuosity across the racial groups (color figure online)

We classified tortuosity as a mild, single loop, and multiple loop formation (Table 2). Mild tortuosity was noted more commonly in the observed cases. At the V1 segment on the left, cases of tortuosity was 22.9% in White, 12.8% in Indian and 6.5% in Black (*p* = 0.058). There was no significant difference across the racial groups. At the V1 segment on the right, a significant difference was noted across the racial groups (21.1% White, 9.2% Indian and 4% Black: *p* = 0.012) (Fig. 4).

**Table 2:** Classification of incidence of tortuosity at the V1 and V2 segments

Tortuosity	Mild (%)	Single loop (%)	Multiple loop (%)	Total (%)
V1 Left side	27.4	13.9	0.9	42.2
V1 Right side	25.5	7.6	1.3	34.4
V2 Left side	9.2	4.5	2.5	16.3
V2 Right side	10.6	2.9	2.3	15.8

**Fig. 5:**
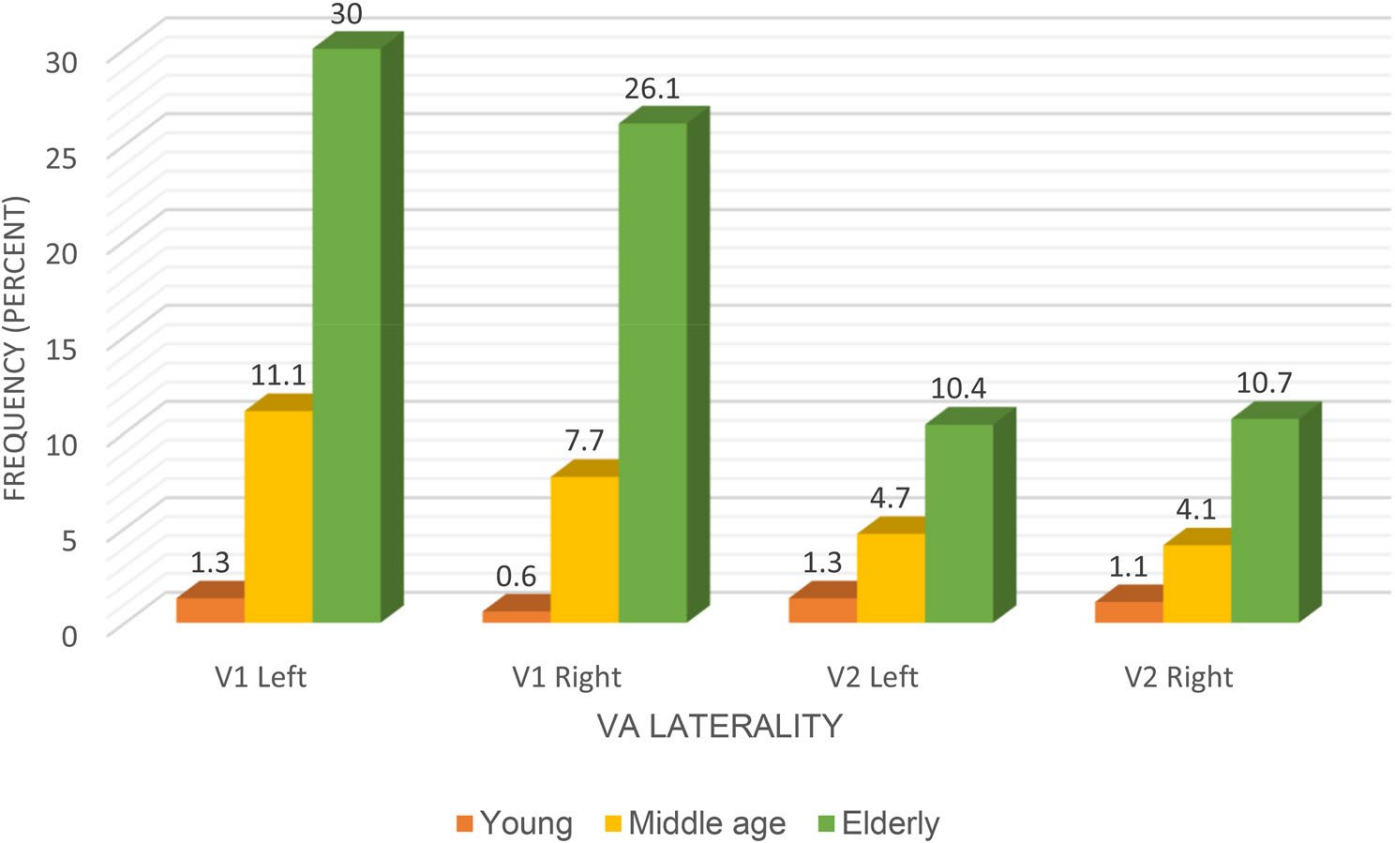
Bar chart of the incidence of tortuosity in relationship with age. We categorized the age as young (10–39), middle age (40–59) and elderly (60–99) (color figure online)

At the V2 segment on the left, differences observed were not significant across the racial groups (7.6% White, 4.2% Indian and 4.5% Black: *p* = 0.093), but the differences were significant on the right (8.6% White, 4.2% Black and 3% Indian: *p* = 0.008). A total of 30% of the cases of tortuosity at the V1 segment on the left is among the elderly patients above 60 years (*p* < 0.001); on the right, 26.1% also for the elderly group (*p* < 0.001). At the V2 segment on the left, 10.4% is among the elderly patients *p* = 0.514 left 10.4% for the elderly (*p* = 0.514), and on the right, 10.7% (*p* = 0.243) (Fig. 5).

### Diameter

The average diameter of the VA in the typical + variation group, the typical group only, and the variation group only at V1 is summarized in Table 3. The average diameter on the left side was significantly larger than the right in all (typical + variation group, typical group *p* = 0.000) except in variation group where we registered significantly larger right than the average diameter on the left side (*p* = 0.011). Considering sex difference, males [right 3.61 (0.78), left 3.69 (0.88)] and females [right 3.61 (0.79), left 3.69 (0.97)] had the same diameter on both sides. The average diameter within the racial groups at the V1 segment is summarized in Table 4.

**Table 3:** Average diameter, length, and laterality of the VA V1 segment in all the cases, variation group only and typical group only

	Typical + variation group morphometries		Variation group morphometries		Typical group morphometries	
	Laterality		Laterality		Laterality	
	Left median (IQR)	Right median (IQR)	Left median (IQR)	Right median (IQR)	Left median (IQR)	Right median (IQR)
Number	554	554	38	38	516	516
Average diameter (mm)	3.69 (0.88)	3.61 (0.79)	3.30 (0.74)	3.78 (0.73)	3.69 (0.88)	3.60 (0.79)
Average length (mm)	42.91 (11.89)	38.22 (11.09)	85.22 (23.82)	44.80 (15.15)	41.97 (11.49)	37.76 (11.08)

Results are reported as median (IQR) in mm

**Table 4:** Average diameter and length of the proximal VAs (V1, V2) and laterality in South African racial groups

	V1		V2	
	Left	Right	Left	Right
Black				
Diameter	3.52 (0.88)^a^	3.43 (0.79) NS	3.43 (0.66)^a^	3.34 (0.79) NS
Length	40.26 (15.26)^c^	35.57 (12.41) NS	59.64 (12.49) NS	59.31 (14.33) NS
Indian				
Diameter	3.82 (0.88)^ab^	3.70 (0.79) NS	3.78 (0.70)^bc^	3.52 (0.71) NS
Length	44.50 (13.18)^c^	39.05 (12.84) NS	62.07 (14.89) NS	59.84 (15.61) NS
White				
Diameter	3.69 (0.79)^a^	3.70 (0.79) NS	3.69 (0.79)^a^	3.52 (0.71) NS
Length	42.53 (10.86) NS	37.97 (10.21) NS	60.37 (10.95) NS	57.86 (11.63) NS

Results are reported as median (IQR) in mm

*Significant difference* a, b, c *p* < 0.05 (Kruskal–Wallis test followed by Wilcoxon Signed Rank test), *NS* No statistical significance

^a^Black vs White

^b^Indian vs White

^c^Indian vs Black

At the V2 segment, the average diameter was significantly larger on the left compared to the right *p *= 0.000 (Table 5). The average diameter within the racial groups at the V2 segment is summarized in Table 4. Considering sex difference, the average diameter in males [right 3.43 (0.71), left 3.60 (0.79)] was not significantly different from that of females [right 3.52 (0.71), left 3.62 (0.88)] (right *p* = 0.250, left *p* = 0.750).

**Table 5:** Average diameter, length, and laterality at the V2 segment

Parameter	LVA median (IQR)	RVA median (IQR)	Wilcoxon sign rank *p* value
Diameter (mm)	3.60 (0.86)	3.52 (0.71)	0.000*
Length (mm)	60.73 (12.24)	58.25 (12.67)	0.000*

Results are reported as median (IQR) in mm

*p* value < 0.05 was considered statistically significant

*LVA* left VA, *RVA* right VA

### Hypoplasia and pattern of dominance

Incidence of VA hypoplasia and pattern of dominance at the V1 and V2 segments are summarized in Table 6. Males had a slightly higher proportion of hypoplastic arteries (4.2%) compared to females (2.9%) on the right side, though this was not significant (*p* = 0.643). On the left side, 3.6% were females, 2.3% were males (*p* = 0.056).

**Table 6:** Incidence of hypoplasia and pattern of dominance at the V1 and V2 segments

VA segment	VAH			VAD		
	Right	Left	Bilateral	Right	Left	Codominance
V1 *n* (%)	39 (7)	33 (6)	5 (0.9)	216 (39.7)	284 (52.2)	44 (8.1)
V2 *n* (%)	43 (7.8)	34 (6.1)	5 (0.9)	206 (37.3)	318 (57.6)	28 (5.1)

*VAD* VA dominance, *VAH* VA hypoplasia

At the V2 segment on the right side, 3.1% cases of hypoplasia were in females, 4.7% in males (*p* = 0.488); on the left side, 2.9% in females, 3.2% in males (*p* = 0.765). Hypoplastic arteries were noted bilaterally in five patients (0.9%) at V1 and V2 segment.

### Length

The average length at the V1 segment in the typical + variation, variation and typical group is summarized in Table 3. The left VA was significantly larger in all the groups (*p* = 0.000).

Considering sex differences, there was no significant difference in the average length of males [right 38.49 (11.89), left 43.37 (12.01)] and females [right 37.92 (10.59), left 42.24 (11.77)]: right (*p* = 0.685), left (*p* = 0.376). The average length within the racial groups at V1 segment is summarized in Table 4.

The average length at the V2 segment is summarized in Table 5. A significant difference was noted between left and right (*p* = 0.029). Considering sex differences, the average length in males [right 59.66 (13.19), left 61.25 (12.46)] was significantly larger on the right compared with the females [right 56.96 (12.03), left 59.84 (11.88)]. Right *p* = 0.021, Left *p* = 0.128. The average length within the racial groups at V2 segment is summarized in Table 4.

## Discussion

**Fig. 6:**
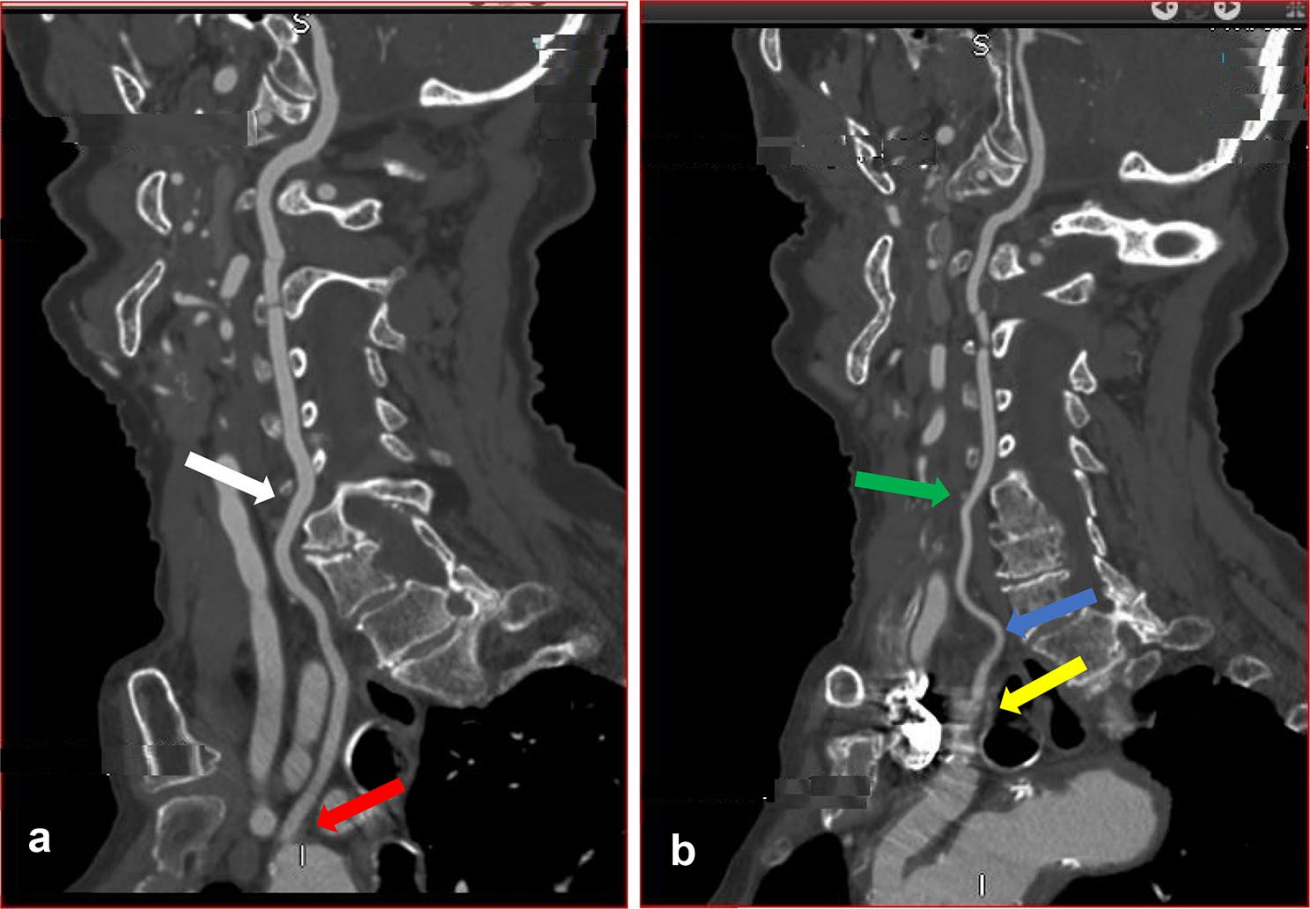
Sagittal view of the left (**a**) and right (**b**) VA of a female patient. **a** The left VA (red arrow) originates directly from the AOA and enters through the transverse foramen of C5 (white arrow). **b** The right VA took origin from the right subclavian artery (yellow arrow) and ascends through the transverse foramen of C4 vertebra (green arrow). The blue arrow shows single medial loop at the V1 segment (color figure online)

**Fig. 7:**
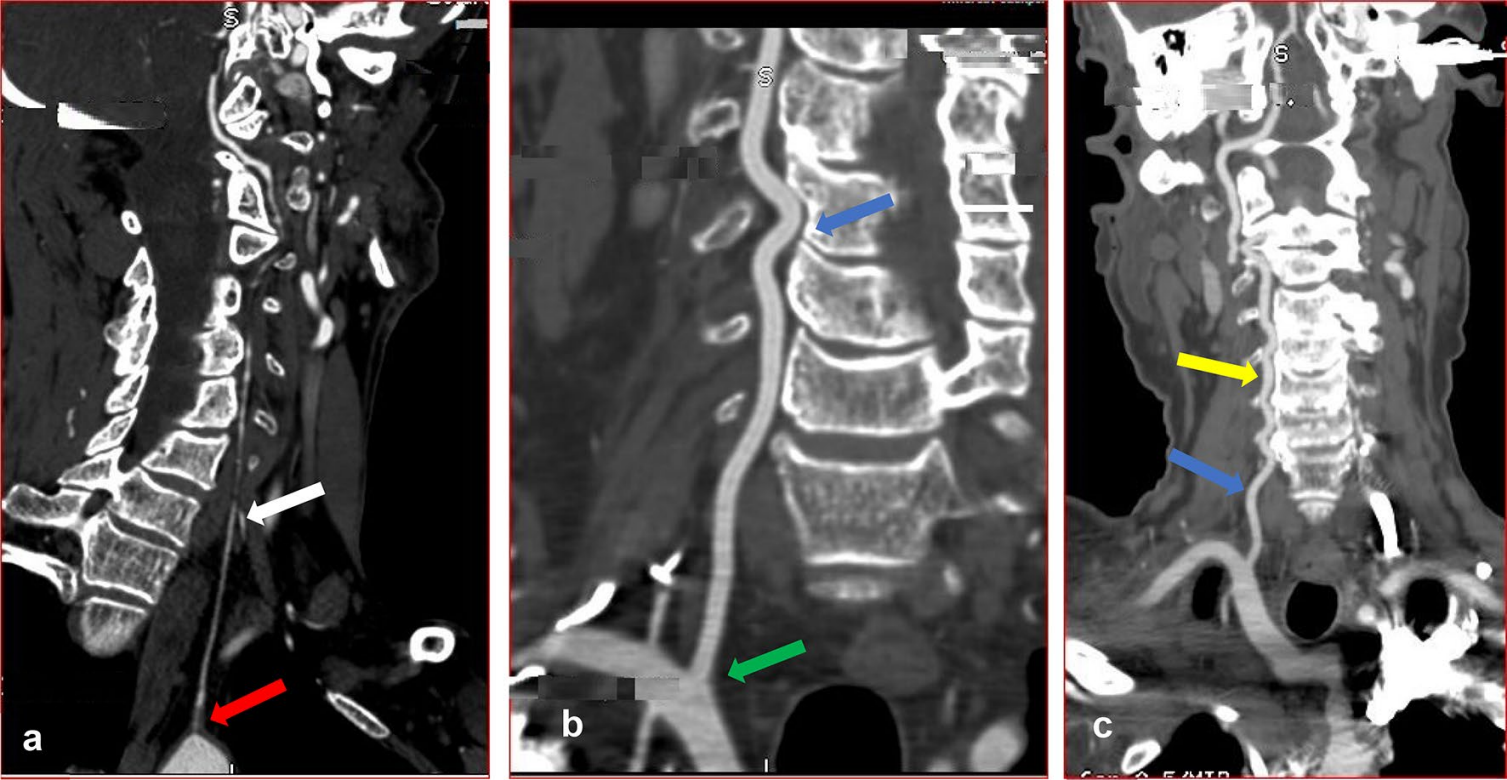
Coronal view of CTA images of a male (**a**, **b**) and female (**c**) patients showing variation in origin, hypoplasia, medial loop at V2 and mild tortuosity at V1 and V2. **a** Red arrow shows origin of the left hypoplastic vertebral artery (white arrow) from the aortic arch. **b** The dominant right VA emanates from the subclavian artery (green arrow) with a single medial loop into the vertebral body of C4 (blue arrow). **c** Shows mild tortuosity at V1(blue arrow) and V2 segments (yellow arrow) of the right VA (color figure online)

Previously, it was suggested that variable origin of the VA did not result in clinical symptoms [21, 27]. However, a recent review of literature has shown that anatomical variations in origin may be symptomatic when they coexist with vascular malformations such as aneurysms, aortic dissection, coarctation, and congenital heart disease [25]. Some researchers have also indicated that knowledge of this variation is crucial for head and neck surgical procedures, angiographies, carotid artery or VA stent placement and during the evaluation of vascular pathologies such as arterial dissection [24, 37]. The most-reported variation in the origin of the VA artery is the emergence of left VA (LVA) directly from the AOA [2, 25]. Our study follows this trend as all variant origins are from the AOA between the left subclavian artery and the left common carotid artery (Fig. 3a, 6a and 7, 2a). The incidence in the current study (6.9%) is similar to previous cadaveric report from South African population (8.5%) (Doctoral dissertation, University of Cape Town). Our result is also comparable to recent reports that used similar imaging modalities (CTA) in an Egyptian population (7%) [1] but slightly higher than reports from Republika Srpska population (4.47%) [40] and Turkish population (3.6%) [2]. In the present study, the comparison of the incidence across the racial groups showed that variation in origin is highest in White (3.6%), followed by Indian (2.5%) and least in Black (0.7%) South African. However, the differences were not significant. All the right VA took their origin from the subclavian artery, although two of the right VA arose from the subclavian artery close to the bifurcation of the innominate artery (brachiocephalic artery) (Fig. 3b). These two VA entered the TF of C4 and C5. The contralateral VA on the left, which originated from the AOA, ascends via the same TF. This type of bilateral variation compares favorably to the study reported by Amgain [3].

Variation in the origin is often associated with other morphological variations, such as the level of entry into the TF. Complete knowledge of typical anatomy and awareness of possible variation are crucial to prevent iatrogenic injuries to the artery. Preoperative information of anatomical variation with regards to the point of entry of the VA is important to reduce the risk of injury during an anterior and lateral approach to the cervical spine [4]. Interestingly, in the present study, all the left VAs that originated from the AOA entered the TF of cervical vertebrae other than that of C6 vertebra except 1 (2.6%) (Table 1). Previous reports have shown that the highest prevalence of anomalous entry is at C5, followed by C4, C7, and the least reported is at C3 [1, 40]. The typical and variation group on both left and right sides in our report follows a similar trend. Even though all the right VAs took origin from the subclavian artery, 9.4% (52) exhibited variation in the level of entering the TF (Fig. 7, Table 1). Our results indicate that variation in the point of entry into the TF is not predictable. It can occur even when the artery has a typical origin. However, it should be suspected on the contralateral right VA when there is variation in origin on the left. In the present study, variation in the point of entering the TF appears common on the right when the left VA originates from the AOA, as shown in Fig. 3a, b.

Fundamental knowledge about the complex embryogenesis of the VA can give some clues to understanding the basis of variation in its morphology. As previously known, the VA is formed around the 32nd day of the embryonic stage (during 7–12 mm embryo stage) by the persistence of multiple longitudinal anastomotic chains between the six (or seven) adjacent cervical intersegmental arteries (CIA) formed from the longitudinal arteries of the aortic trunk [14]. According to George and Bruneau, each of these intersegmental arteries is a potential site of arterial agenesis that could result in morphologic variation (George and Bruneau 2011). During the stage of fusion and anastomosis, complications could result in various vascular anomalies like the variation in origin and point of entering the foramina transversarium, tortuosity, hypoplasia, and asymmetry right and left VA as observed in the present study. We hypothesized that complications might set in due to some genetic and environmental factors.

The site of origin of the VA depends on the CIA that persists at the adult stage to become the prevertebral segment, which is usually the sixth CIA [35]. A possible explanation for variation in origin from the AOA, as observed in the present study (Fig. 2a, 3a, 6a and 7a), is the persistence of a single primitive CIA other than the sixth [13]. The persistence of two primitive CIAs simultaneously may duplicate the origin of the VA [13]. However, we did not observe any case of duplicate origin in the present study. Some researchers have suggested that the persistent vessel can be identified by its level of entrance into the TF rather than by its origin [5, 13]. For instance, a VA formed by persistent 5th CIA always enters the C5 TF although may originate from the subclavian artery, AOA, or other previously reported variant origins. According to this theory, anomalous entry of the VA into the TF reported at C3, C4, and C5 in the present study is due to persistent 3rd, 4th, and 5th CIAs. We also report on the point of entry into the TF of the C7 vertebra, but it is unclear which CIA persisted at this level for its occurrence.

Normally, the course of the VA at V1 and V2 segments is straight. However, due to anatomical variation, VA sometimes demonstrates slight loops in all directions. These can result in a significant and potentially dangerous medial or a lateral artery displacement known as tortuosity. VA tortuosity is an uncommon congenital or acquired anomaly mostly reported at the proximal VAs (V1, V2) and rarely reported at the distal part of the artery. The embryogenesis of tortuous VA is not clear [18]. It is also difficult to assess the suggested congenital origin, according to some authors, since vascular tortuosity increases with age [2]. Morris et al. reported that vertebrobasilar artery tortuosity develops in association with connective tissue disorders [32]. Other authors postulate reduced elasticity, degeneration of blood vessels, and vascular wall shear stress as a possible cause [18, 26]. Another study suggested that vascular risk factors (such as hypertension, diabetes, and lipid metabolism disorders) may promote atherosclerosis, aging, and blood vessels' degeneration, thereby aggravating the vertebrobasilar artery tortuosity [20]. However, there is no conclusion on the mechanism of formation. In the present study, tortuosity occurs most frequently at V1 in elderly patients (Fig. 5). We hypothesized that the weakness of the connective tissue that makes up the vascular wall due to aging might contribute to the formation of a tortuous vessel. Also, its frequency at the V1 segment may be because this part of the artery is unconfined while the TF supports the V2 segment in its entire course. Some researchers have suggested that progressive medial deviation of loops of the tortuous VA at the V2 segment (as shown in Fig. 2b 7b) may erode the vertebral body and further cause TF enlargement and nerve root entrapment [2, 4]. A wide range of neurovascular problems has been diagnosed in association with loop formation, as reported in the literature [10, 19]. In contrast, another study suggested that VA loops and tortuosity are asymptomatic and incidentally diagnosed during the evaluation of neck problems and trauma [7]. This information suggests that VA tortuosity may be a co-factor for the clinical symptoms mentioned above and not the primary cause. The prevalence of VA tortuosity has been previously reported in the literature. A Turkish population sample of 35 patients reported 78.3% and 21.6% tortuosity at V1 and V2, respectively [42]. A Japanese study found only one medial loop at V2 out of 1054 patients [41], while a similar study reported 13.6% cases of tortuosity in 110 patients at the V2 segment [2]. Other authors reported no incidence of tortuosity in their population [28]. Most of these reports are from the Asian continent. Incidence of tortuosity seems to exist at a dissimilar rate in different regions, and data from the African population are scarce. In the present work, we report 76.6% and 32.1% tortuosity at the V1 and V2, respectively. This finding is consistent with the previously mentioned report from the Turkish population. Based on our findings, the incidence across the racial groups was similar on the left but significantly high in White on the right at V1 and V2 (Fig. 2c 7c). A possible explanation for the disparity in the rate of occurrence of tortuosity in various populations, as mentioned above, and across the South African racial groups, may be differences in the genetic make-up coupled with other environmental factors. Previous study has shown that tortuosity increases with age [2], and this is probably why it is sometimes described as an acquired anomaly. In agreement with this observation, our result shows that the incidence of tortuosity is common in elderly patients (age range 60–99 years). Tortuosity predisposes the VA to iatrogenic risk during instrumentation procedure especially in corpectomy [2, 8]. In the present study, series of mild, medial, and lateral single; and multiple loops (Figs. 3a, 6b, 7b, c, 2b, c) were seen most at the V1 and less frequently at the V2 segment. Our results indicate that incidence of tortuosity is high in the studied group and should be cautiously look out for during preoperative planning.

Noticeably in the variation group, the VA’s width at the V1 segment is significantly larger on the right compared to the left (Table 3). We hypothesized that the left VA of AOA origin frequently has a reduced diameter or sometimes hypoplastic while the right VA is likely to be the dominant artery. A recent anatomic study on Chinese cadavers observed similar significantly larger right VA compared with the left VA in a group with variant LVA origin [28]. This morphologic variation should be considered during the endovascular intervention and preoperative planning around the V1 segment when the origin of the LVA is from the AOA. According to a pathology textbook, 80% of people have a left dominant VA [15]. This theory has also been corroborated by most authors reporting larger left VA in the literature. Our study supports this trend as the average diameter at the V1 segment is similar to that of the Egyptian population (3.67 ± 1.07, 3.36 ± 0.93) [1], with the left significantly larger than the right side. A consistent significantly larger left was also observed at the V2 segment in the present study. This information is essential during preoperative planning: iatrogenic injury to the dominant VA can result in death [17]. Cervical spine surgery or screw fixation is best carried out on the right side of the cervical vertebrae in this situation so that the left VA can sustain the blood flow in the vertebrobasilar system in case of iatrogenic injury to the right VA.

A condition where the lumen diameter of the VA is exceptionally small in its entire length and terminates at its fusion with the contralateral artery to form the basilar trunk is described as VA hypoplasia (VAH) (Fig. 6a, 7a) [15]. VAH is a frequent morphological variation with a history of underestimated clinical significance. Probably because it is a common finding in asymptomatic population [22]. However, accumulating evidence has shown that VAH is commonly observed in patients with posterior circulation stroke and VA occlusion [22, 31]. This suggests that VAH can predispose to posterior circulation stroke. This may be the possible explanation for the high prevalence of VAH reported by studies using suspected stroke and acute ischemia patients 15.6% [38], and 31.5% [31]. There is no consensus as regards the diameter value for VAH. Authors have used criteria ranging from ≤ 2 mm to ≤ 3 mm [11, 12, 28].

In the present study, a diameter < 2.7 mm is regarded as hypoplastic, as previously described by [12]. Ergun et al. defined VAH using a ≤ 2 mm criterion found an incidence of 7.1% on the right and 9.4% on the left in patients examined by digital subtraction angiographies [11]. In a CT study by Abd El Gawad et al. In a CT study by Abd El Gawad et al. VAH was defined by a diameter ratio of 1:2 between the two VAs and found an incidence rate of 8% on the left, and 3% on the right between the two VAs found an incidence rate of 8% on the left, and 3% on the right [1]. Our findings showed the number of patients with hypoplastic VA on the right at V1 was 7% (39 individuals) and 6% (33 individuals) on the left. At the V2 segment on the right, there was a slight increase in the number of patients, 7.8% (43 individuals), while the number is similar on the left, 6.1% (33 individuals). The prevalence in our study is consistent with the above report by Abd El Gawad et al. using similar imaging protocols and patient characteristics.

Several factors contribute to the challenges of selecting a universal cut-off value of VAH for the general population. The average VA diameter recorded in the literature varies because most of the studies report on different segments of the artery. The differences in the modality of the studies can also contribute to the reported disparity in the average diameter. A recent report has shown a strong correlation between the diameter of the VA and anthropometric parameters like height [12]. All these factors and suspected genetic and environmental factors may be the basis for the differences reported in the literature. The incidence of bilateral hypoplasia in the present study is observed only in 5 patients (0.9%) at V1 and V2. Our findings indicate that the dominant artery can be preserved in most cases during instrumentation procedures since VAH is rare bilaterally.

Vertebral artery dominance (VAD) is a congenital structural variation of the VA characterized by a significant diameter difference between bilateral VAs of the same individual. The prominent VA is now described as the dominant. The embryogenesis of this morphologic variation is not clear, just like that of the VAH. Some researchers hypothesized differences in the origin of bilateral VAs as the possible cause of left dominant VA [29, 44]. The left VA being a second branch of the aorta, while the right VA is the third branch. However, this theory fails to explain the mechanism of the formation of right dominancy. VAD was previously described as mere structural variation without clinical relevance until recently. Researchers now identify VAD as a risk factor for posterior circulation infarction, brainstem infarction, and transient ischemic attack [29, 44].

Scholars have adopted numerous criterion for the definition of VAD in the literature, and till present, there was no agreement on the standard criteria. A recent study using magnetic resonance angiography used a side difference of ≥ 0.3 mm and found left VA to be dominant in 63.6% [43] patients. Other researchers used a size difference of ≥ 1.2 mm and showed similar results in 56.4% of patients with similar image modalities [44]. Ozdemir et al. adopted a standard of VAD using any size difference criteria between the bilateral VAs and observed 64% left dominancy and 31% right dominance with color Doppler ultrasound [34]. As mentioned above, the studies’ results were consistently similar even with different dominance criteria. Based on the criteria of any size difference, the VA width in the present study was evaluated as left dominant in 52.2% (V1), 57.6% (V2), right dominant in 39.7% (V1), 37.3% (V2), and codominance in 8.1% (V1), 5.1% (V2) (Table 6). Our result is consistent with the previous report as we report left VAD in most patients. Therefore, it is possible for physicians performing procedures around the VA to preserve the dominant VA, which is expected on the left side in most cases. However, a case-by-case review of preoperative investigations may be required for interventions in the vicinity of the VA as well as for diagnosis of pathologies in the posterior circulatory territory. This is due to the unpredictable nature of variation as noticed in our report.

In the present study, the average length of the variation group at the V1 segment was almost twice the length of the right. This is obviously because all the left VAs in this group emanated directly from the AOA. However, the average length of the left VA in the typical group was also significantly larger than the right. A possible explanation for the side-to-side differences observed in the typical group is the asymmetric origins of the right and left subclavian arteries [39]. However, this theory is yet to be proven and requires further investigation. Few anatomical studies have documented the average length of the first segment of the VA in the literature. Veeramani and Shankar reported a significant difference between left (4.1 ± 1.7 cm equivalent to 41 mm) and right (3.4 ± 1.2 cm equivalent to 34 mm) in 33 Indian cadavers [39]. Our findings are similar to the above study report, as we observe average length 42.91 (11.89) mm and 38.22 (11.09) mm on the left and right VA, respectively. Similar to the V1 segment, the average length of the left VA at the V2 segment is significantly larger than the right (Table 5). We assumed that the above-suggested reason for the V1 segment might have positioned the entire length of the left VA higher than the right.

Generally, because of the unpredictable nature of anatomical variation, the physician performing surgery around the proximal VA needs to be aware of safe technique and standard anatomy and possible anatomical variations [16]. Opinion differs about the necessities of routine preoperative angiographies for evaluating cerebral vasculature due to its downsides such as ionizing radiation and iodinated contrast [33]. While some authors propose CTAs before cervical spine interventions [2], others have suggested that it is unnecessary, especially when the prevalence of anatomical variations of the VAs is low in a population group [33]. The importance of in-depth knowledge of VA anatomy in patients undergoing cervical spine surgery cannot be overemphasized. This is required for a detailed analysis of the preoperative radiograph [8]. Due to morphological variations and frequencies of tortuosity reported in the population in the present study, we hypothesized that CTA might be required for safe surgical interventions around the VA and endovascular treatment of vascular pathologies.

### Study limitation

Firstly, this is a single-center retrospective study with the possibility of selection bias. Secondly, specific information about the interracial difference was not provided in the patient's report. Therefore, the authors relied on the available information in the hospital database to deduce patients' race since the study is retrospective.

## Conclusion

Anatomical variations of the VA are common in the South African population studied in this work. All variant origin was observed on the left without any significant racial or gender difference. Variation in the point of entry into the TF is similar on both sides. Tortuosity is common in the V1 segment and frequently observed in elderly patients. The average diameter was significantly larger on the left in all the racial groups, but there were no significant gender differences. Hypoplasia occurs at a similar rate on both left and right, and we registered a left dominance pattern. The presence of these morphological variations can influence treatment options for procedures in the head and neck, and regions of the supra-aortic arch. Awareness of the extent of possible anatomical variation will help in interpreting radiographs which will enhance identification of vascular pathologies and will also reduce the risk of iatrogenic injury.
